# Correlation of telomere length and DNA damage markers with severity and associated depressive states in patients with OSAS

**DOI:** 10.3389/fneur.2025.1633137

**Published:** 2025-10-20

**Authors:** Yu Jiang, Meixin Qu, Feng Cheng, Huifen Sun, Jie Dai, Zhen Wu

**Affiliations:** Department of Otorhinolaryngological, Affiliated Changshu Hospital of Nantong University, Changshu, China

**Keywords:** telomere length, DNA damage markers, OSAS patients, severity, depressive state, correlation

## Abstract

**Objective:**

Investigation of the correlation of peripheral blood leukocyte relative telomere length (RTL) and DNA damage markers with severity and related depressive states in patients with obstructive sleep apnea syndrome (OSAS).

**Methods:**

A total of 110 OSAS patients admitted to the hospital between January 2023 and December 2024 were selected. Based on examination results, the OSAS patients were divided into the mild to moderate and the severe groups. Additionally, 45 healthy volunteers who voluntarily participated in the study during the same period were included as a healthy control group. RTL and DNA damage markers [phosphorylated histone H2AX (γH2AX), 8-hydroxydeoxyguanosine (8-OHdG)] of the three groups were compared among the three groups. The Hamilton Depression Scale (HAMD) was used to assess the depressive status of OSAS patients, who were further categorized into the depressed group (HAMD score >7) and non-depressed group (HAMD score ≤7) based on the severity of depression. The correlation of RTL and the above markers with the severity of the disease of OSAS patients and their HAMD scores was analyzed by *Pearson* correlation analysis, with the efficacy of RTL and DNA damage markers in predicting secondary depression in OSAS patients analyzed by plotting the receiver operating characteristic curve (ROC).

**Results:**

The RTL values of patients in the severe and the mild to moderate group were significantly lower than those of the healthy control group with higher levels of γH2AX and 8-OHdG, and the differences of three indexes between the severe group and the mild to moderate group registered statistical significance (*p* < 0.05). *Pearson* analysis showed that RTL was negatively correlated with the HAMD scores of patients with OSAS (*r*1 value = −0.65); γH2AX and 8-OHdG were positively correlated with the HAMD scores of OSAS patients (*r*2 value = 0.62, *r*3 value = 0.65, both *p* < 0.05). ROC analysis based on RTL and DNA damage markers found that the area under the curve (AUC) values of RTL, γH2AX, and 8-OHdG for predicting secondary depression in OSAS patients were 0.665, 0.865, and 0.752, respectively.

**Conclusion:**

RTL, γH2AX, and 8-OHdG exert an important role in assessing the severity of disease in OSAS patients. Besides, there exists a significant correlation between the three indexes and the depressive state of patients, boasting a good predictive efficacy for their secondary depression.

## Introduction

Obstructive sleep apnea syndrome (OSAS) is a common sleep-related breathing disorder characterized by repeated partial or complete collapse of the upper airway during sleep, leading to episodes of apnea and hypopnea. This pathological process arises from the interaction of multiple factors, including anatomical abnormalities of the upper airway (such as pharyngeal fat accumulation due to obesity or mandibular retrognathia), impaired neuromuscular regulation, and reduced tension of the pharyngeal dilator muscles during sleep. These factors collectively contribute to airway obstruction, triggering a cascade of physiological responses. Patients with this condition often experience arousal from sleep and daytime sleepiness. As the disease progresses, it may lead to declines in memory and cognitive function, changes in personality, reduced libido, and pose serious risks to overall health and wellbeing (1–3). Epidemiologic investigations have found that the prevalence of OSAS in the adult population is as high as about 20% and about 90% of these patients fail to make a definitive diagnosis and active treatment, registering a low rate of early diagnosis of OSAS (4). The relaxation of the soft tissues around the oropharynx or the muscles of the respiratory tract during sleep is easy to block the upper airway, which may result in the obstruction of respiratory gas exchange, thus leading to the organism being in a state of intermittent hypoxia (prolonged intermittent hypoxia can trigger neurological damage) (5). It has been discovered that OSAS is closely related to the occurrence of various diseases such as cerebrovascular diseases, mental abnormalities, pulmonary heart disease and respiratory failure (e.g., OSAS increases the risk of developing Alzheimer’s disease in patients, which may be connected with the imbalance of neurotransmitter systems in the brain caused by sleep deprivation, giving rise to the impairment of memory pathways and inefficient signaling of synaptic structures); such severe secondary morbidity may even result in the loss of consciousness of patients, which leads to not only a steep decline in their quality of life, but also greatly increases the risk of death (6, 7). Therefore, it is important to assess the severity of OSAS patients at an early stage and actively carry out targeted treatment to improve the prognosis and quality of life of patients.

Polysomnography (PSG) monitoring is the “gold standard” for diagnosing OSAS and assessing its severity. By simultaneously recording multiple physiological signals such as electroencephalogram (EEG), electrooculogram (EOG), electromyogram (EMG), respiratory airflow, blood oxygen saturation, and electrocardiogram (ECG), PSG provides a comprehensive evaluation of sleep architecture, respiratory events, degree of hypoxia, and cardiac rhythm changes (8). According to international guidelines, the apnea-hypopnea index (AHI) is the core parameter interpreted from PSG and is used to quantify the severity of the disease. However, due to poor compliance among some patients, who may find it difficult to cooperate with PSG monitoring, obtaining actual examination results can be challenging. This, in turn, affects physicians’ ability to accurately assess the severity of OSAS and hinders the subsequent implementation of treatment (9, 10). Then, serologic tests, cytologic tests and other diagnostic modalities have been proposed in the clinic. The change of relative telomere length (RTL) of peripheral blood leukocytes is a major discovery in the study of human aging mechanisms. In recent years, this index has been widely used in the study of the occurrence and development of various diseases (e.g., diabetes, uremia, cancer, etc.); for example, some scholars have detected that the shortening of telomeres is correlated with the occurrence and severity of diabetes; the length of telomeres in tumor cells of patients with cancer has been significantly shortened, which can reflect the malignancy and invasiveness of tumors (11, 12). Relevant experiments have pointed out that there is a close connection between RTL and hypoxia status in human body, and this index can be used as a diagnostic basis for hypoxia and clinical treatment indication, which leverages a certain guiding role in the evaluation of OSAS condition (13).

OSAS is far from being a simple issue of nighttime snoring; it is a serious systemic disease whose harm stems from recurrent intermittent hypoxia and fragmented sleep architecture during sleep (14). Hypoxia is widely recognized as one of the most critical pathophysiological features of OSA. It inflicts multifaceted damage on the body (including poor sleep quality, cardiovascular diseases, metabolic disorders, etc.) by triggering oxidative stress, systemic inflammatory responses, and sympathetic nervous system excitation (15). This chronic intermittent hypoxia can lead to significant carbon dioxide retention, stimulating the excessive production of reactive oxygen species (ROS) and resulting in intracellular DNA damage (16). DNA damage is prevalent in OSAS patients, and DNA damage markers [e.g., phosphorylated histone H2AX (γH2AX), 8hydroxydeoxyguanosine (8-OHdG)] are expected to participate in the assessment of OSAS disease severity (17). This study focused on analysis of the correlation of RTL and DNA damage markers with the severity and related depression status of OSAS patients, aiming to provide a reasonable reference basis for the early and accurate clinical assessment for the severity and secondary depression status of OSAS patients.

## Data and methods

### Research objects

This study was a single-center prospective observational analysis, 110 OSAS patients admitted to the hospital from January 2023 to December 2024 and 45 healthy controls were selected. The study was approved by the Ethics Committee of our hospital and all procedures were conducted in accordance with the ethical standards of the 1964 *Declaration of Helsinki* and its subsequent amendments.

### Inclusion and exclusion criteria

Inclusion criteria: (1) Patients in the OSAS group met the diagnostic criteria for OSAS as outlined in the *Society of Anesthesia and Sleep Medicine Guidelines on Preoperative Screening and Assessment of Adult Patients With Obstructive Sleep Apnea* (18). Diagnosis was confirmed based on an AHI ≥ 5 calculated from PSG monitoring results, combined with clinical manifestations (such as loud and irregular snoring, sensations of choking during sleep, daytime sleepiness, difficulty concentrating, etc.) and imaging examinations (X-ray or CT revealing airway collapse or structural abnormalities); (2) Over 18 years old; (3) Not combined with other diseases that cause RTL abnormalities or DNA damage; (4) With the *Informed Consent Form* signed; (5) No mental illness.

Exclusion criteria: (1) Patients with combined malignant tumors; (2) Patients with contraindications of examinations related to this study; (3) Patients accompanied by severe dysfunction of major organs such as the heart, liver, or kidneys; (4) Patients with comorbid hereditary DNA damage repair deficiency disorders or telomere biology abnormalities (e.g., dyskeratosis congenita, Bloom syndrome, etc.), active systemic infections, autoimmune diseases (such as systemic lupus erythematosus, rheumatoid arthritis, etc.), hematological diseases (e.g., lymphoma, leukemia, etc.), recent major surgery or trauma, or a history of radiation or chemical exposure that may affect RTL or DNA damage were excluded; (5) Patient with other chronic hypoxic diseases, such as chronic obstructive pulmonary disease, interstitial lung disease, etc.; (6) Patient with severe neurological diseases or cognitive dysfunctions; and (7) Females being pregnant or lactational.

### General conditions

Basic information of all subjects were obtained based on relevant questionnaires, including gender, age, history of underlying diseases [diabetic patients met the diagnostic criteria in the *2019 ESC Guidelines on diabetes, pre-diabetes, and cardiovascular diseases developed in collaboration with the EASD* (19)] and hypertensive patients all met diagnostic criteria in the *Blood pressure and the new ACC/AHA hypertension guideline* (20), smoking history (smoking ≥100 cigarettes within the past 1 year), drinking history (frequency of drinking ≥1 in a week for 5 consecutive months) and body mass index (BMI) [BMI = weight (kg)/height squared (m^2^)].

### Sleep breathing parameters

All subjects received PSG monitoring with a polysomnographic EEG recorder (model: TREX HD, Nicorette Instruments, Inc., USA) used. The monitoring period was from 9:00 p.m. in the evening to 6:00 a.m. of the next day; patients were instructed to abstain from drinking, smoking, coffee, strong tea, sedative and psychotropic medications for 24 h before administration of the PSG. The monitoring items included electroencephalography, ocular motility, oral and nasal airflow, mandibular electromyography, oxygen saturation and thoracic and abdominal respiratory movement with the AHI values effectively recorded. All the sleep apnea parameters required for this trial were audited and generated in assistance by the same trained technician.

### Laboratory tests

Fasting peripheral venous blood samples of 4 mL were collected from all subjects and placed in vacuum blood collection tubes; blood samples were promptly sent for examination with centrifugation operations were carried out for 15 min at room temperature (1,900 r/min^−1^) to obtain serum and plasma samples. The serum and plasma samples were stored at a low temperature (−80 °C) for testing.

Routine blood tests: instrument: five-classification blood cell analyzer (model: BC5300, Shenzhen Myriad, China), laboratory testing personnel strictly followed the operating procedures of all blood specimens to carry out routine blood tests and recorded the number of red blood cells, neutrophil count and hemoglobin concentration. The blood sample testing operation was completed within 4 h of delivery.

Blood glucose testing instruments: open-type automatic biochemical analyzer (model: RocheModular P800, Roche, Switzerland); automatic glycated hemoglobin analyzer (model: HLC-723G7, TOSOH, Japan); the content of fasting blood glucose (FBG) and glycated hemoglobin (HbAlc) in serum samples was tested; FBG detection method: glucose oxidase method; HbAlc detection method: high performance liquid chromatography. The blood sample testing operation was completed within 4 h of delivery.

RTL detection was performed according to the method described in reference (21). The instrument used was a quantitative fluorescence PCR system (model: TOptical, Jena, Germany), and the reagent kit employed was the MagicSYBR Mixture quantitative fluorescence kit (Baichuan Biotechnology, China). After the peripheral venous blood samples were thawed, leukocyte DNA was isolated and extracted; telomere length was quantified by the Cawthon’s real-time fluorescent quantitative polyribonucleotide chain reaction (qPCR) technique, with the t/s ratio were computed to generate values related to the average telomere length by comparing the amount of telomere amplification product (t) to the amount of single-copy genes (s). Primers were designed and synthesized by Liuhe UW Genetics Technology Co. with the upstream primer for telomeres being 5′-CGGTTTGTTTGGGTTTGGGTTTGGGTTTGGGGTTTGGGTTTGGGGTTT-3′ and the downstream primer 5′-GGCTTGCCTTACCCTTACCCTTACCCTTACCCTTACCCT-3′. The human control gene primers were HBG1: GCTTCTGACACAACTGTGTTCACTAGC and HBG2: CACCAACTTCATCCACGTTCACC. Each sample was assayed in triplicate, and the reaction program was set up with the qPCR reaction system: 95 °C, 5 s for pre-denaturation and then implemented 40 cycles of 60 °C for 1 min, 95 °C for 15 s and 60 °C for 1 min.

Detection of the DNA damage marker 8-OHdG was performed according to the method described in reference (22). The instrument used was a fully automated enzyme labeling instrument (Model: Varios-kan LUx, Thermo Fisher Scientific, USA), and the reagent kit employed was the human 8-OHdG enzyme linked immunosorbent assay (ELISA) kit. Laboratory inspectors are interested in detecting the level of serum 8-OHdG in strict accordance with the instructions of the kit, Detection method: ELISA. The blood sample test operation was completed within 4 h of delivery.

Detection of the DNA damage marker γH2AX was performed with reference to related literature (23) using an immunofluorescence method. Respiratory epithelial cells in logarithmic growth phase were inoculated into 6-well cell culture plates in which slides had been placed with the cells transfected with different vectors, respectively, for 48 h, followed by cell fixation (4% paraformaldehyde) for 30 min, and then washed with phosphate-buffered saline solution (PBS) with 0.3% Triton-X 100 added afterwards. The cells were permeabilized for 30 min, washed 3 times and 5 min/time with PBS; then closed with 5% bovine serum protein sealing solution for 1 h. Next, rabbit anti-γH2AX antibody (1:200) was added overnight at 4 °C and washed with PB; then sheep anti-rabbit fluorescent secondary antibody (1:200) was added to the chamber for 1 h; PBS was washed 3 times and 5 min/time, ensued with addition of 4′,6-diamidino-2-phenylindole (DAPI) solution and incubation in the dark for 10 min with Subsequent washing by PBS for 3 times, and finally added appropriate amount of anti-fluorescence bursting agent dropwise for observation of the picking diagram using fluorescence microscope.

### Statistical treatment

In this paper, data were analyzed by IBM SPSS 27.0 (IBMCorp., Armonk, NY, USA) statistical software, and count data were expressed by [n] with the x2 test adopted; measurement data were carried out by the Shapiro–Wilk method for the test of normal distribution: those that did not conform to the normal distribution were expressed using the median and interquartile spacing [M (P25, P75)], while those exhibiting conformity to the normal distribution were expressed as ( $\bar{x}\pm s$ ) followed by the t-test with the difference was considered statistically significant at *p* < 0.05. The correlation between RTL and DNA damage markers of severity and associated depressive states in OSAS patients was analyzed by *Pearson* correlation analysis with linear correlation modeled by linear regression; GraphPad Prism 8.3 was used to perform the statistical analysis with test level: *α* = 0.05 and predictive efficacy was evaluated by subject work characteristics (ROC) curves.

## Results

### Flowchart

**Figure 1:**
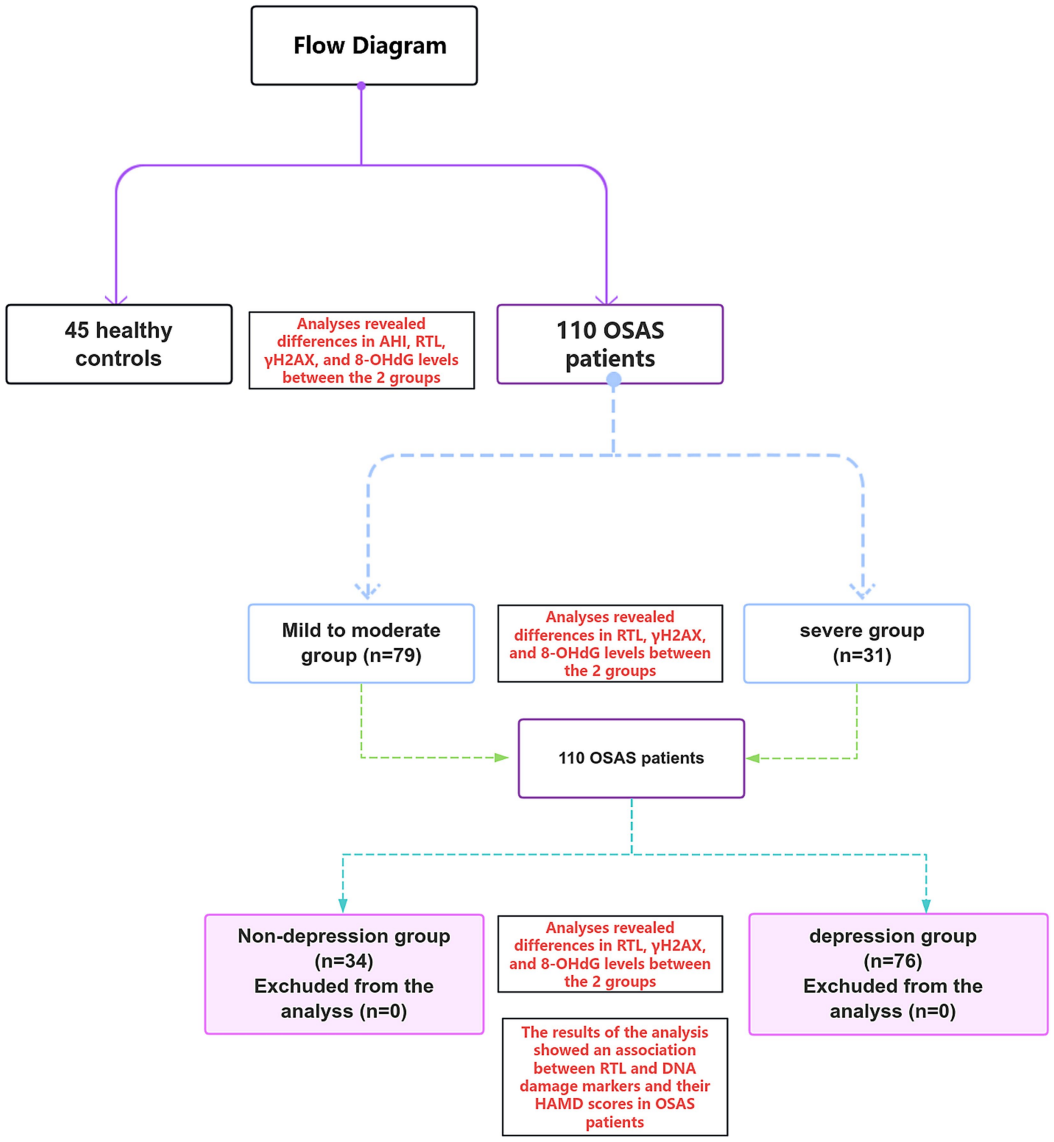
Flowchart of specific grouping and parameter comparative analysis in this study.

The detailed flowchart of group allocation and parameter comparative analysis in this study is shown in Figure 1.

### General data and laboratory tests

#### General data

There was no statistical difference between the healthy control group and the OSAS group in terms of gender, age, average duration of disease, BMI, smoking history, drinking history and history of underlying diseases (all *p* > 0.05, Table 1).

**Table 1:** Comparison of general data in the two groups.

Terms		Healthy control group (*n* = 45)	OSAS group (*n* = 110)	*χ^2^*/Z	*p*
Gender (cases)	Male	21 (53.33)	72 (65.45)	1.977	0.160
	Female	21 (46.67)	38 (34.55)		
Age [years, M (P25, P75)]		47.00 (44.00, 56.00)	49.00 (45.80, 54.00)	−0.575	0.565
Average duration of disease [months, M (P25, P75)]		7.55 (5.90, 8.80)	7.56 (6.10, 9.20)	−1.041	0.298
BMI [kg·m^−2^, M (P25, P75)]		26.56 (24.20, 28.50)	26.24 (24.00, 27.90)	−0.822	0.411
Smoking history		9 (20.00)	20 (18.18)	0.106	0.745
Drinking history		7 (15.56)	18 (16.36)	0.015	0.901
History of underlying diseases	Hypertension	10 (22.22)	24 (21.82)	0.003	0.956
	Diabetes	8 (17.78)	19 (17.27)	0.006	0.940

#### Laboratory tests

The erythrocyte count (5.29 ± 1.03 × 10^12^/L vs. 5.41 ± 1.05 × 10^12^/L), neutrophil count (6.38 ± 1.94 × 10^9^/L vs. 7.05 ± 2.56 × 10^9^/L), hemoglobin concentration (158.76 ± 17.84 g-L^−1^ vs. 156.09 ± 16.33 g-L^−1^), FBG (4.15 ± 1.09 mmol-L^−1^ vs 4.39 ± 0.98 mmol-L^−1^) and HbAlc (6.04 ± 1.17%vs. 6.39 ± 1.20%) levels were compared, with the no significant difference (all *p* values > 0.05); in comparison with the healthy control group, AHI values of patients in the OSAS group were obviously higher (4.72 ± 0.93 times·h^−1^ vs 34.97 ± 6.87 times·h^−1^) with statistical significance displayed (*p* < 0.05, Table 2).

**Table 2:** Comparison of laboratory tests and sleep parameters of the two groups.

Terms	Healthy control group (*n* = 45)	OSAS group (*n* = 110)	*t*	*p*
Erythrocyte count (×10^12^/L)	5.29 ± 1.03	5.41 ± 1.05	0.649	0.517
Neutrophil count (×10^9^/L)	6.38 ± 1.94	7.05 ± 2.56	1.579	0.116
Hemoglobin concentration (g·L^−1^)	158.76 ± 17.84	156.09 ± 16.33	0.899	0.370
FBG (mmol·L^−1^)	4.15 ± 1.09	4.39 ± 0.98	1.339	0.183
HbAlc (%)	6.04 ± 1.17	6.39 ± 1.20	1.660	0.099
AHI (times·h^−1^)	4.72 ± 0.93	34.97 ± 6.87	29.372	<0.001

### Association of RTL and DNA damage markers with disease severity in OSAS patients

#### Comparison of RTL and DNA damage markers in healthy controls and OSAS patients with different severity of disease

**Figure 2:**
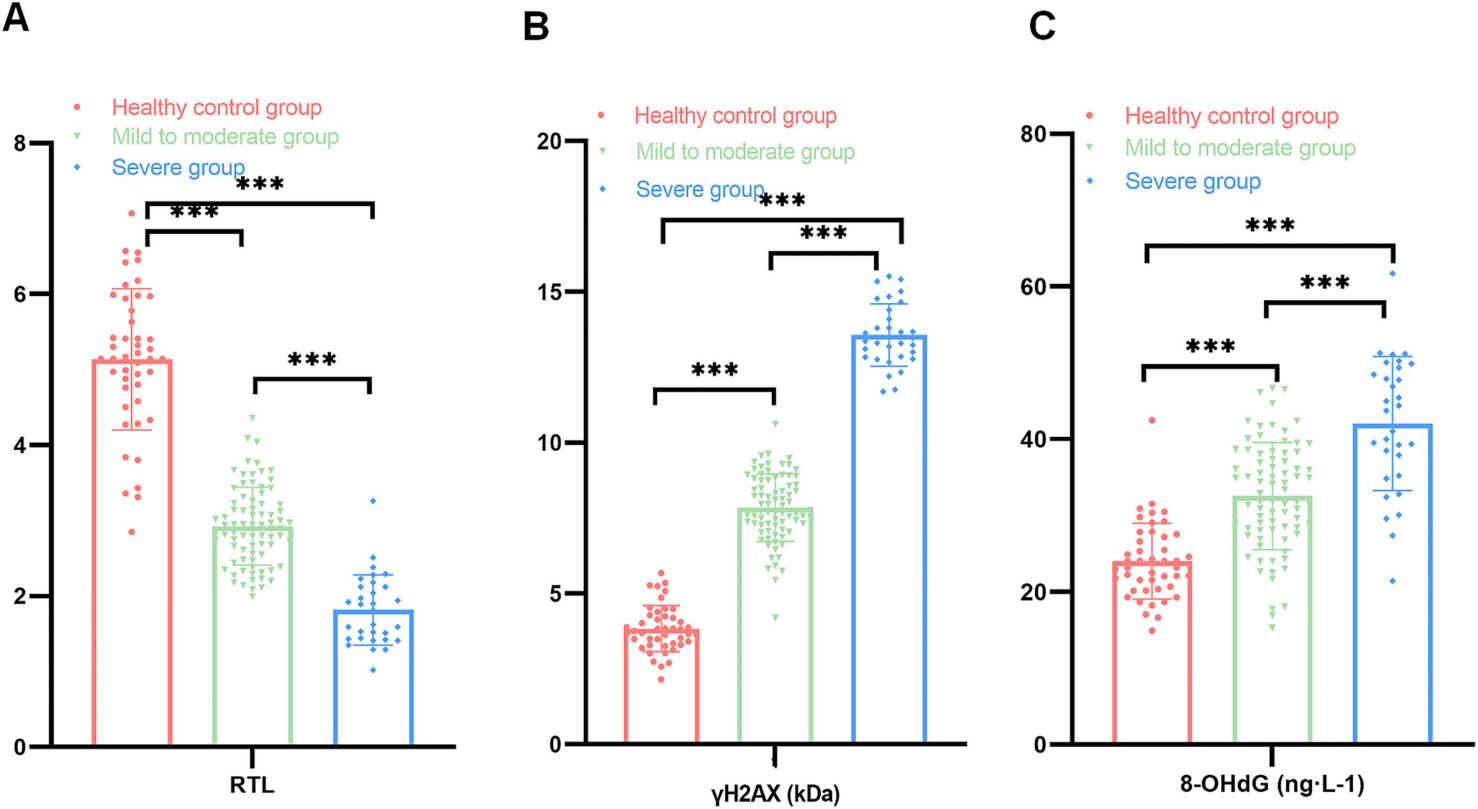
Comparison of RTL and DNA damage markers among the three groups (**A**: comparison of RTL among the healthy control group, the mild to moderate and severe groups; **B**: comparison of γH2AX among the healthy control group, the mild to moderate and severe groups; **C**: comparison of 8-OHdG among the healthy control group, the mild to moderate and severe groups; ***p < 0.001).

Patients in the OSAS group were divided into the mild to moderate group (79 cases) and the severe group (31 cases) as per AHI values, and levels of RTL and DNA damage markers were compared between the above two groups and the healthy controls; as exhibited in Figure 2, the RTL values of the patients in the severe and mild to moderate groups were clearly lower than those of the healthy control group with levels of γH2AX and 8-OHdG being higher, and the differences were statistically significant (*t*1 value = 17.189, *t*2 value = 21.361, *t*3 value = 7.159, *t*4 value = 18.360, *t*5 value = 47.094, *t*6 value = 11.388, *p* < 0.05); the comparison of RTL values and γH2AX and 8-OHdG levels between patients in the mild to moderate group and those in the severe group indicated statistical significance (*t*7 value = 10.491, *t*8 value = 24.529, *t*9 value = 5.942, *p* < 0.05, Figure 2). Such being the case, the above data suggested that there was a correlation between RTL and DNA damage markers and disease severity in OSAS patients.

#### Correlation analysis of RTL, DNA damage markers and sleep breathing parameters in OSAS patients

**Figure 3:**
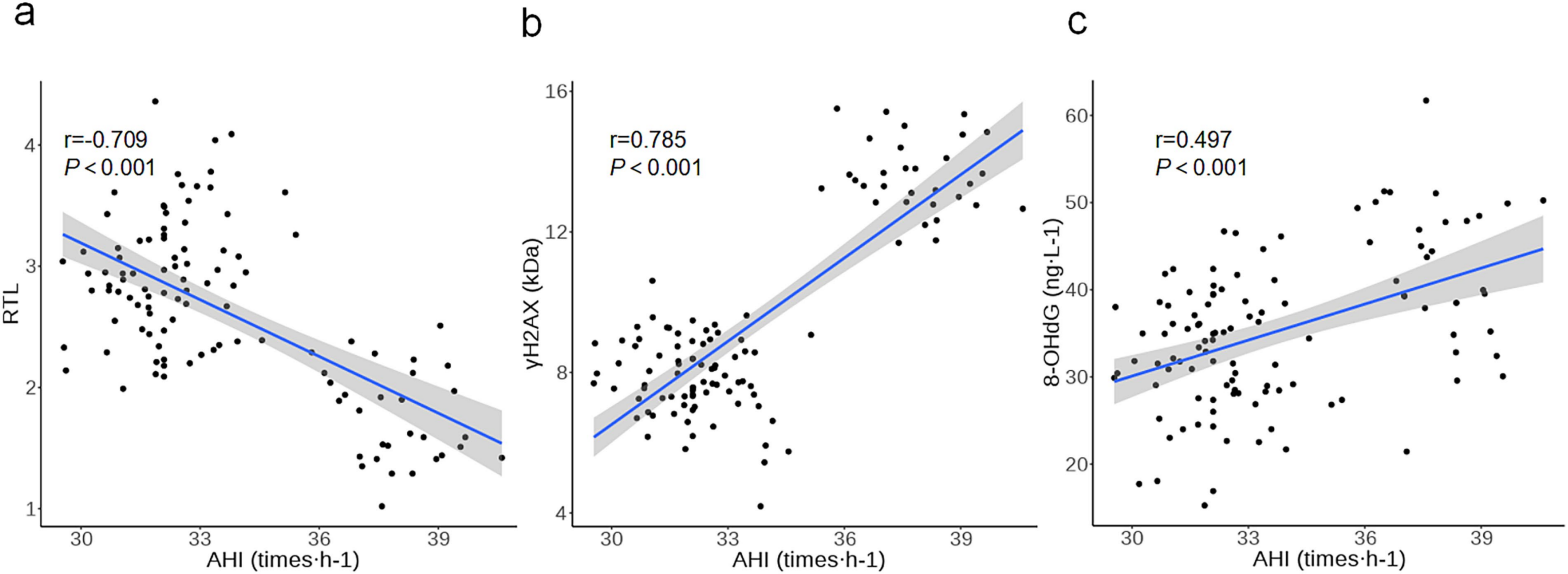
Correlation between RTL, DNA damage markers and sleep breathing parameters in OSAS patients (**a**: correlation between RTL and AHI values in OSAS patients; **b**: correlation between γH2AX and AHI values in OSAS patients; **c**: correlation between 8-OHdG and AHI values in OSAS patients).

*Pearson* correlation analysis revealed that AHI values of OSAS patients were positively correlated with γH2AX and 8-OHdG (*r* = 0.785, 0.497, *p* < 0.001) whereas negatively correlated with RTL values (*r* = −0.709, *p* < 0.001), as displayed in Figure 3, which implied that RTL and DNA damage markers were related to AHI values of OSAS patients and the indicators might influence each other and participate in the disease progression of OSAS patients.

### Association of RTL and DNA damage markers with related depressive states of OSAS patients

#### Evaluation of related depressive states in OSAS patients

All patients in the OSAS group were surveyed with the Hamilton Depression Scale (HAMD) to assess depressive status; 76 patients with a total HAMD scale score over 7 were included in the depression group with 34 patients below 7 included in the non-depression group.

#### Comparison of RTL and DNA damage marker levels between patients in depressed and non-depressed groups

Compared to the non-depression group, patients in the depression group showed a significant decrease in RTL values with their γH2AX and 8-OHdG levels registered apparently higher, exhibiting a statistical significance (*t*1 = 3.196, *t*2 = 8.083, *t*3 = 6.478, *p* < 0.05, Table 3).

**Table 3:** Comparison of RTL and DNA.

Targets	Non-depression group (*n* = 34)	Depression group (*n* = 76)	*t*	*p*
RTL	2.05 ± 0.62	1.69 ± 0.51	3.196	0.002
γH2AX (kDa)	9.17 ± 2.03	11.92 ± 1.45	8.083	<0.001
8-OHdG (ng·L^−1^)	36.07 ± 8.94	43.56 ± 3.17	6.478	<0.001

#### Correlation of RTL and DNA damage markers with related depressive states of OSAS patients

**Figure 4:**
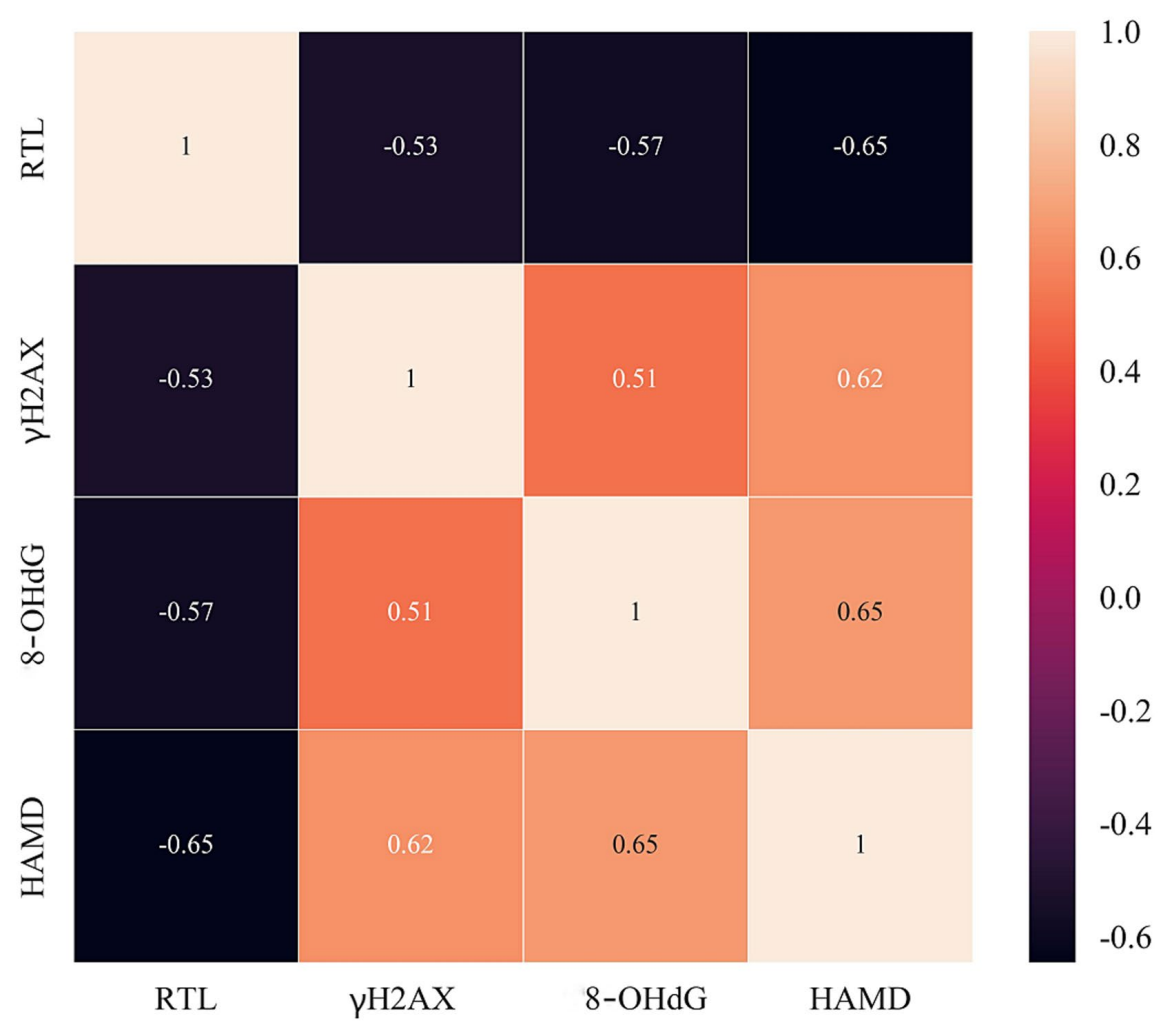
Correlation between RTL, DNA damage markers (γH2AX, 8-OHdG) and HAMD scores in OSAS patients.

*Pearson* correlation matrix analysis indicated that RTL and DNA damage markers (γH2AX, 8-OHdG) were significantly linearly correlated with HAMD scores in OSAS patients, with the results of the visual correlation analysis displayed in Figure 4; furthermore, a remarkable linear negative correlation was detected between RTL and HAMD scores in OSAS patients with *r* value = −0.65; γH2AX, 8-OHdG and HAMD scores of OSAS patients checked a measurable linear positive correlation, with *r* values = 0.62 and 0.65, respectively. Such results indicated that RTL and DNA damage markers had a close correlation with related depressive state in OSAS patients with a high degree of correlation.

### Efficacy of RTL and DNA damage markers to predict secondary depression in OSAS patients

**Figure 5:**
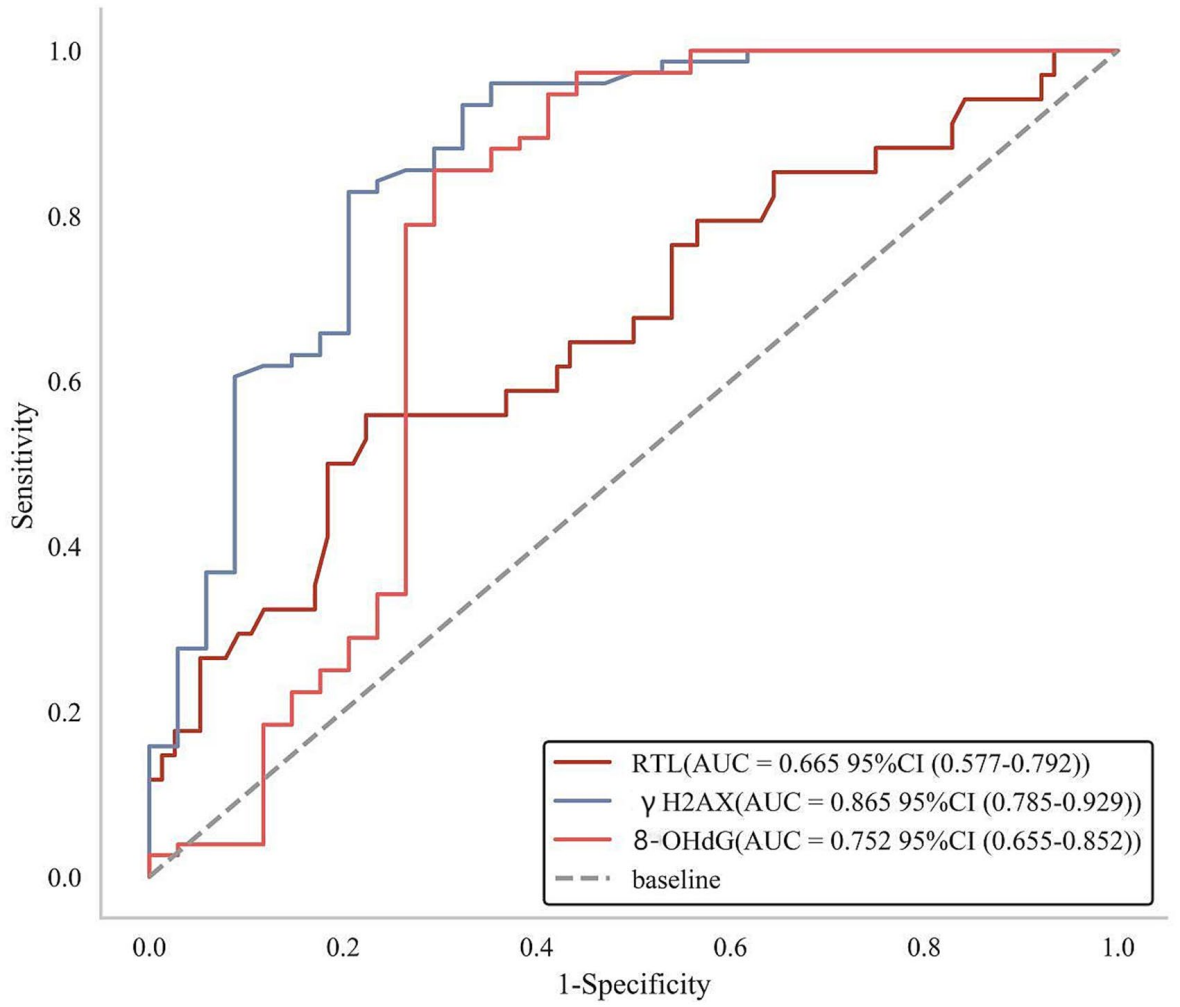
ROC curves of RTL and DNA damage markers to predict secondary depression in OSAS patients.

According to the ROC curves, it was found that the AUC value of RTL to predict secondary depression in OSAS patients was 0.665 (95% CI: 0.577–0.792), with the AUC value of γH2AX as 0.865 (95% CI: 0.785–0.929) and the AUC value of 8-OHdG as 0.752 (95% CI: 0.655–0.852), which manifested that all the above three indexes boasted good predictive value for secondary depression in OSAS patients (Figure 5).

## Discussion

In OSAS patients, the occurrence of intermittent upper airway obstruction cause recurrent apnea, decreased blood oxygen saturation and tissue hypoxia during sleep. This process generates excessive oxygen free radicals, disrupting oxygen balance and triggering oxidative stress (24, 25). Currently, the treatment methods for OSAS constitute a multi-level and systematic framework. According to the recommendations of the *Clinical Practice Guideline for Diagnostic Testing for Adult Obstructive Sleep Apnea: An American Academy of Sleep Medicine Clinical Practice Guideline* (26), continuous positive airway pressure (CPAP) is the first-line standard and effective treatment for patients with moderate to severe OSAS. Substantial evidence-based medical research has demonstrated that adherent use of CPAP can significantly eliminate respiratory events, correct intermittent hypoxia, improve sleep architecture, alleviate daytime sleepiness symptoms, and positively intervene in cardiovascular comorbidities such as hypertension. For patients with mild OSAS or those intolerant to CPAP therapy, oral appliances (OA) serve as an important alternative, particularly suitable for positional cases or those without severe hypoxia. For patients who fail conservative treatments like CPAP or OA and have clear correctable upper airway anatomical abnormalities (such as significant tonsillar hypertrophy, severe nasal septum deviation, mandibular retrusion, etc.), surgical intervention is often considered the final treatment strategy, aiming to expand upper airway volume and relieve anatomical obstruction.

Recent studies have revealed that OSAS has a certain genetic predisposition. Genome-wide association studies (GWAS) have identified multiple genetic loci associated with the risk of OSA onset, and the occurrence of this disease is linked to certain genetic polymorphisms. Conditions such as bruxism and temporomandibular joint disorders (TMJ) also exhibit significant familial aggregation and genetic tendencies, sharing potential genetic mechanisms with OSA. Notably, the prevalence of OSAS is significantly higher in patients with genetic syndromes such as Down syndrome and Treacher Collins syndrome compared to the general population. This hereditary craniofacial structural abnormality is closely associated with the pathogenesis of OSAS (27). Thus, genetic factors play a central role in the pathophysiology of OSAS. It has been shown that oxidative stress triggered by chronic intermittent hypoxia can induce DNA strand breaks and oxidative DNA damage, leveraging an impact on the stability of chromosome structure (28). DNA double-strand breaks (DSBs) is one of the most severe types of DNA damage, which mainly refers to the simultaneous breakage of two complementary strands of the DNA double-helix structure at the same counterpart or in close proximity to each other; the breaks induce the formation of γH2AX by phosphorylation of serine at position 139 of the conserved region of histone H2AX (29, 30). Relevant studies have discovered that a large amount of γH2AX (in a characteristic punctate structure) can be observed under the microscope in a short period of time (1–3 min) after the occurrence of DSBs in cells, and the expression of this substance reaches a peak around 10 min after the occurrence of DSBs, which indicates that γH2AX is involved in the process of DNA damage response and such indicator is an important marker for evaluating the occurrence of DSBs in cells (31). 8-OHdG is an important marker of oxidative DNA damage, which reflects the metabolic status of the body and cellular repair capacity; the toxic effects of ROS exert a damaging effect on human tissues, causing oxidative DNA damage and triggering apoptosis (32). In this study, the content of γH2AX and 8-OHdG in the severe group presented significantly higher than that in the mild to moderate group, which appeared statistical significance with a positive correlation between the AHI value and γH2AX and 8-OHdG in OSAS patients. Such data indicated that the degree of oxidative damage in OSAS patients gradually increased with the progression of the disease, resulting in a more pronounced situation of DNA damage in the cells of the organism. Therefore, early detection of DNA damage markers makes contributions to accurate determination of the severity of OSAS for clinicians.

In addition, researchers have found that peripheral blood lymphocytes from OSAS patients also suffer from oxidative DNA damage leading to a reduced cellular ability to repair damage. Chromosomal damage in peripheral blood lymphocytes is closely related to telomeres (33). Telomeres are specialized sequences composed of telomeric DNA and telomere-binding proteins, mainly located at the ends of eukaryotic linear chromosomes, which provide good protection for chromosomes by forming t-loop compact structures or binding to telomere-protecting proteins in order to prevent end fusion (34). It has been found that telomere length can be gradually shortened as somatic cells continue to proliferate, and when telomeres shorten to a certain extent, cells stop dividing (35). Hence observation of the changes in RTL values makes a certain guiding sense in evaluation of the cellular damage status. In this study, it was found that the RTL value of the severe group represented visibly lower than that of the mild to moderate group, which was negatively correlated with the AHI value of OSAS patients. The reason for that may be that hypoxia has a mediating effect on the activation of telomerase, and the hypoxemia and hypercapnia triggered by apnea in OSAS patients may accelerate the depletion of telomeres, thus contributing to the apoptosis of cells. Therefore, telomere length change is expected to be a measurable observational index for assessment of the degree of hypoxia in OSAS patients. It is noteworthy that smoking and alcohol consumption are clinically recognized as significant external factors contributing to oxidative stress and inflammation. Such adverse lifestyle habits may lead to telomere shortening or exert detrimental effects on DNA integrity. Additionally, metabolic and vascular disorders such as diabetes and hypertension are themselves major drivers of accelerated aging and may similarly influence telomere dynamics. Furthermore, biological principles indicate that females generally exhibit longer average relative telomere length (RTL) compared to males of the same age. In this study, statistical analyses were conducted to account for these potential confounding variables. The results revealed no statistically significant differences between the OSAS group and the healthy control group in terms of smoking, alcohol consumption, diabetes, hypertension, or gender distribution. This lack of observed association may be attributed to the relatively small sample size of the study, which limited statistical power. Moreover, the effects of risk factors such as smoking, alcohol use, and underlying chronic conditions may be cumulative or intermittent, often manifesting at low doses that are challenging to detect clinically. Consequently, the analysis failed to identify small to moderate effect sizes, resulting in non-significant comparative outcomes.

OSAS poses the risk of asphyxiation, and patients are prone to negative emotions due to excessive worry about their condition. Relevant surveys have shown that the number of OSAS patients with depressive symptoms can range from 18 to 57% (36). Depression not only increases the risk of neurocognitive impairment in OSAS patients in conjunction with hypoxic injury, but may also bring about loss of treatment confidence and decreased adherence, thus negatively affecting clinical outcomes (37). As a result, it is necessary to predict the depressive state of OSAS patients at an early stage and take timely intervention measures. The data of this study showed that compared with the non-depression group, the RTL value of patients in the depression group decreased significantly with the levels of γH2AX and 8-OHdG registering prominent increasement, which indicated that patients with OSAS secondary to depression showed significant changes in RTL and DNA damage markers with the specific mechanism may be the more appeared oxidative stress damage in the body of patients accompanied by high expression of 8-OHdG after the onset of OSAS. Besides, 8-OHdG induces oxidative stress in nerve cells by activation of oxygen radicals and hydroxyl radicals and effects on the normal metabolism of nerve cells, thus damaging neuronal tissues and promoting the occurrence of depression. Moreover, chronic psychological stress developed in OSAS patients by disease and other factors accelerates cellular senescence, leading to a generalized shortening of factors closely related to cellular senescence (i.e., telomeres) (38). In this study, ROC analysis revealed that RTL, γH2AX and 8-OHdG possessed high AUC values for predicting secondary depression in OSAS patients, which meant that the three indicators were potential biomarkers for determining secondary depression in OSAS patients.

This study represents the first comprehensive evaluation of the combined role of RTL and DNA damage markers (γH2AX, 8-OHdG) in the severity of OSAS and depressive status, demonstrating significant potential for clinical translation. However, several limitations should be acknowledged: For instance, while strict matching of baseline characteristics and reasonable control of potential confounding factors (such as smoking, alcohol consumption, diabetes mellitus, hypertension, and gender) were implemented to mitigate bias, larger sample sizes are still needed for further validation. The study lacks longitudinal data, and the objectivity of analysis remains incomplete (e.g., the primary mechanisms underlying depression secondary to OSAS were not thoroughly elucidated). Additionally, other factors that may impact the accuracy of the results were not fully considered, such as potential age-related variations in telomere length measurements among OSAS patients, and the relatively subjective and limited assessment tools used for evaluating depressive status. Therefore, future clinical studies should consider incorporating objective indicators from multiple dimensions, adopting more authoritative assessment tools, conducting multicenter research, and performing comprehensive analyses to actively exclude confounding factors. This approach will ensure the accuracy of results and provide deeper insights into correlation between RTL, DNA damage markers, and the severity of OSAS as well as associated depressive states.

## Conclusion

In summary, RTL and DNA damage markers in OSAS patients registered abnormal changes, coupled with close relation of changes in RTL, γH2AX, and 8-OHdG to disease severity. The mentioned three indexes exerted an important role in assessing the severity of the disease in OSAS patients. Furthermore, RTL, γH2AX, and 8-OHdG also expressed significant correlation with the state of depression in OSAS patients, boasting favorable potential in the prediction of secondary depression in OSAS patients.

## Data Availability

The original contributions presented in the study are included in the article/supplementary material, further inquiries can be directed to the corresponding author.
